# Dynamic ultrasound reveals the specific behavior of the medial meniscus extrusion in patients with knee osteoarthritis

**DOI:** 10.1186/s12891-023-06361-6

**Published:** 2023-04-10

**Authors:** Yosuke Ishii, Masakazu Ishikawa, Yuko Nakashima, Takato Hashizume, Saeko Okamoto, Yoshitaka Iwamoto, Kaoru Okada, Kazuya Takagi, Makoto Takahashi, Nobuo Adachi

**Affiliations:** 1 Department of Biomechanics, Graduate School of Biomedical and Health Sciences, Hiroshima University, 1-2-3 Kasumi, Minami-Ku, Hiroshima, 734-8553 Japan; 2 Department of Orthopaedic Surgery, Faculty of Medicine, Kagawa University, Kagawa, Japan; 3 Department of Musculoskeletal Ultrasound in Medicine, Graduate School of Biomedical and Health Sciences, Hiroshima University, Hiroshima, Japan; 4 Ultrasound Business Operations, Healthcare Business Headquarters, KONICA MINOLTA, INC, Chiyoda-ku, Tokyo Japan; 5 Department of Orthopaedic Surgery, Graduate School of Biomedical & Health Sciences, Hiroshima University, Hiroshima, Japan

**Keywords:** Medial meniscus extrusion, Ultrasonography, Dynamic evaluation, Gait, Knee osteoarthritis

## Abstract

**Background:**

In the dynamic condition, knee osteoarthritis (OA) usually presents with pain. In the weight-bearing condition, a medial meniscus extrusion (MME) may cause severe symptoms and pathological progression. However, the correlation between a dynamic MME and pain has not been elucidated. Now, an MME can be evaluated under dynamic conditions and reflect the characteristics of symptomatic knee OA. This study investigated MMEs during walking and their correlation with knee pain.

**Methods:**

Thirty-two symptomatic patients with knee OA (mean age, 60.5 ± 9.9 years) were enrolled in this study. The medial meniscus was evaluated using ultrasonograms during walking, and in the static supine and unipedal standing positions, as dynamic and static conditions, respectively. The ΔMME (the difference between the maximum and minimum MMEs) was obtained in each condition. The intensity of the knee pain during walking was measured by the visual analog scale (VAS).

**Results:**

The ΔMME in the dynamic condition was significantly higher than that in the static condition (*P* < 0.01). There was a significant correlation between VAS and ΔMME only in the dynamic condition.

**Conclusions:**

The dynamic evaluation is a valid tool for understanding the mechanisms of knee pain and the behavior of the medial meniscus in symptomatic knee OA.

## Background

Patients with medial knee osteoarthritis (OA) have medial knee pain, which is symptomatic under dynamic conditions, such as walking. Additionally, knee pain is known to be associated with abnormal mechanical stress according to the pathological structure [1].

The meniscus plays a role in the distribution of loading stress and its function is known as the hoop function. However, the meniscus gradually shows tears and degeneration due to pathological conditions. A medial meniscus extrusion (MME) presents according to the pathological changes in the meniscus and leads to increased contact pressure on the tibiofemoral joint of the knee [2–4]. The behavior of an MME under loading stress is a feature of knee OA and its reaction indicates the loss of hoop function according to the abnormal mechanical stress [5, 6]. The behavior of an MME is a potential target factor for the detection of mechanical stress, while its correlation with knee pain remains controversial [7–9].

Ultrasonography can be used to observe the behavior of the meniscus under different conditions [6, 8, 9]. In several previous studies, static evaluations, i.e. the difference in the MME between supine and standing positions, were used to calculate meniscus behavior [8–12]. However, the amount of mechanical stress depends on movement, such as standing and walking. A previous study reported that the static condition has poor mechanical stress compared with that under dynamic conditions, such as walking [13]. This indicated that the static evaluation might underestimate the reaction of the meniscus in the daily activities of life. The meniscus has been observed recently during walking. The behavior of the MME in patients with knee OA was evaluated using ultrasonography with a specific probe [14]. This dynamic evaluation might provide more information about the mechanism of knee pain according to the pathological structure.

This study aimed to investigate the MME during walking and its correlation with knee pain. We hypothesized that the dynamic evaluation of the behavior of the MME would yield more information compared to that of the static evaluation, as reflected by knee pain.

## Methods

### Participants

From April 2020 to July 2022, 43 participants diagnosed with primary knee OA were enrolled. The inclusion criteria were as follows (1): the ability to walk without any support, (2): knee pain in a medial compartment of the knee during daily activity for at least 3 months (3): age＞40 years, (4): magnetic resonance imaging (MRI). Their knee OA severity and lower limb alignment were determined radiographically using the Kellgren–Lawrence (K/L) score and femorotibial angle (FTA). The exclusion criteria were as follows (1): history of surgical treatment, trauma, or neurological disorder, (2): valgus knee alignment (FTA＜174), (3): severe knee OA (K/L stage = 4), (4): corticosteroid or platelet-rich plasma injections in the last 6 months.

Finally, thirty-two participants with unilateral or bilateral tibiofemoral knee OA (mean age, 60.5 ± 9.9 years; males, n: 13) were involved in this cross-sectional study. The knee with the most intense pain and greatest severity was chosen if the participant had bilateral knee OA.

### Ethics statement

This study was approved by the Ethical Committee for Epidemiology of the University (Approval Number: E449-4). The study was conducted in accordance with the Declaration of Helsinki.

### Evaluation of the meniscus quality

The menisci tears and locations were evaluated using the MRI images obtained 3 months after the first consultation.

At least two consecutive images that revealed a communicated signal intensity line or its extension into the intra-meniscal area were needed to confirm an abnormal meniscus. The types of tears were divided according to the method used in previous studies [2, 15]. Horizontal tears were defined as linear signals into the meniscus separating the upper and lower parts of the meniscus. Longitudinal tears were defined as vertical lines parallel to the circumferential meniscus. Two or more tears were categorized as complex tears.

In particular, a medial meniscus posterior root tear (MMPRT) was defined as a radial tear located in the posterior root section. It has been described as the ghost sign or giraffe neck sign in previous studies [16, 17].

### Ultrasound evaluation of the meniscus

Based on previous studies [8, 14], the evaluation of the medial meniscus was performed under dynamic and static conditions. The ultrasound device (SNiBLE, KONICA MINOLTA, Japan), with a novel 3–11 MHz special linear-array transducer, was used to evaluate the extruded meniscus. The longitudinal transducer was placed over the medial joint space and the triangular medial meniscus appeared as an echogenic structure between the medial femoral condyle and the tibial plateau. The chosen landmark on the image was clear visualization of the boundary between the medial meniscus and the medial collateral ligament.

**Fig. 1:**
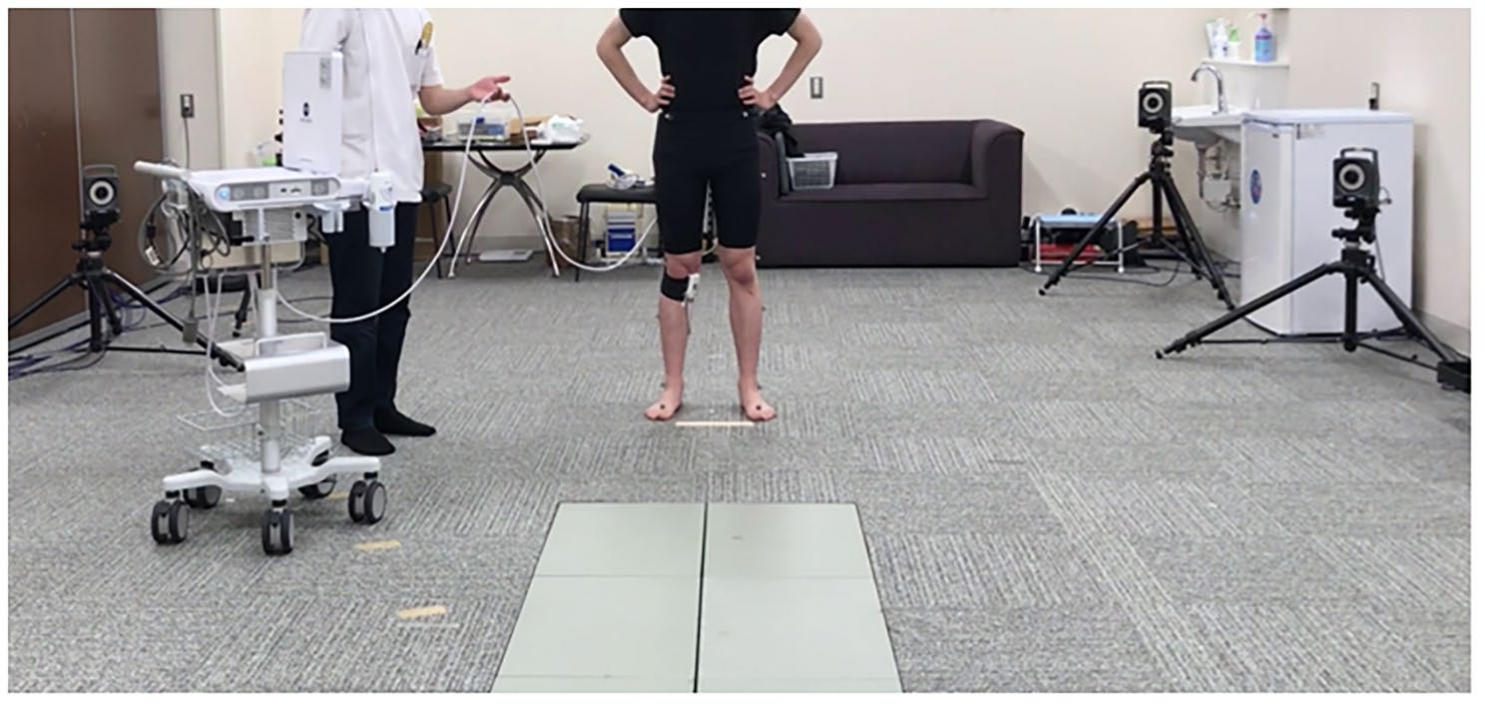
The combination of the ultrasonography and motion analysis systems

In the dynamic condition, the transducer was attached with a flexible band which allowed knee flexion and comfortable walking. The image during walking was recorded in video mode with a sampling rate of 30 Hz. This ultrasonography measurement was demonstrated with a motion analysis system, including motion capture and force plate, simultaneously (Fig. 1). Kinovea software (v0.8.15; Kinovea open source project, www.kinovea.org) was used to adjust the data with different sampling frequencies. Based on a previous study [18], the strain on the ultrasonography image and the peak value of sum acceleration in each plane were determined as the starting points when the examiner touched a dummy marker on the specific transducer. Moreover, a motion video camera (Bonita, Vicon, USA) with a sampling rate of 100 Hz recorded these processes to confirm whether the timing of starting points matched those of the actual actions. This method was shown to be highly reliable in a previous study [18], in which the intraclass and interclass correlation coefficients (ICC 1,3 and ICC 2,3) were 0.89 and 0.78, respectively.

Under static conditions, the transducer was operated and fixed by the examiner’s hands to adjust for appropriate images in different positions, which were based on the previous study [6]. This process demonstrated two positions, supine, and unipedal standing, and the images were recorded three times in each position.

### Gait analyses

The kinematic data and the ground reaction force (GRF) during walking were obtained by a three-dimensional motion analysis system (VICON612; Vicon Motion Systems, Oxford, UK) with sixteen cameras (Vicon Motion Systems), and eight force platforms (AMTI, Watertown, Mass). The sampling rates were 100 and 1000 Hz as cameras and force platforms, respectively. The cameras were calibrated to minimum error and to determine the orientation axis. According to the Plug-in-Gait Marker Set (Plug-in-Gait, Vicon® Peak; Vicon Motion Systems), passive reflex markers were placed on landmarks of the bodies of participants. They were then instructed to walk for 5 m at a comfortable speed at three different times.

On data analysis, the 4th order low pass Butterworth filter with a cutoff frequency of 6 Hz (Vicon® Peak; Vicon Motion Systems) was used to filter the raw data. The knee adduction moment was adopted as an indicator of mechanical stress in the medial compartment of the knee. Additionally, the walking speed and cadence were calculated by anterior-posterior coordinates on their heel markers. These data were selected as a single stance phase in the gait cycle, which was identified from heel-contact to toe-off on the ipsilateral leg. These gait events were determined by the threshold of vertical GRF 10 N.

### Assessment of knee pain

We evaluated the intensity of the knee pain during walking using the visual analog scale (VAS), where a high value represented severe pain. The evaluation was performed immediately after the participants finished walking.

### Calculation of the medial meniscus extrusion

**Fig. 2:**
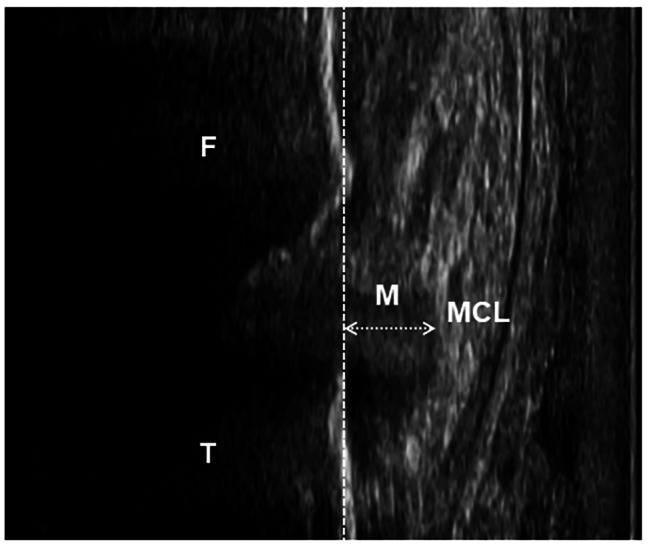
Representative images of the extruded menisci. F: femur, T: tibia, M: medial meniscus, MCL: medial collateral ligament. The dashed line and arrow show the cortex of the medial tibial plateau and the distance as the value of the medial meniscus extrusion

The MME was defined as the distance between the cortex of the medial tibial plateau and the outermost edge of the medial meniscus, according to a previous study [12] (Fig. 2) and was calculated using Kinovea software.

**Fig. 3:**
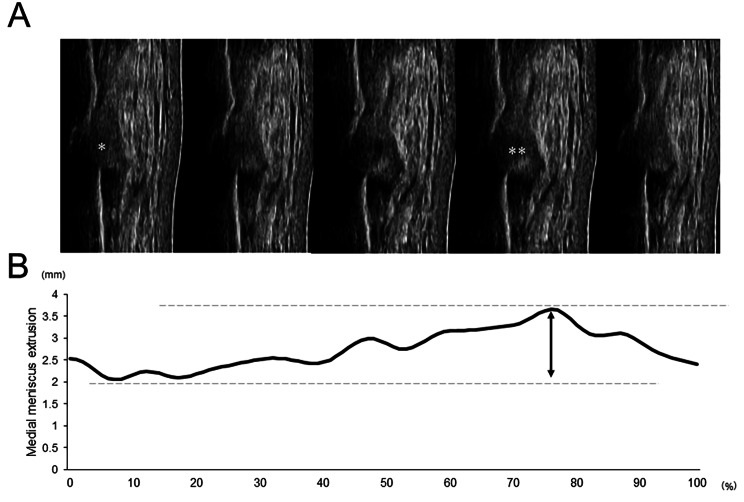
Representative images of the extruded meniscus during the stance phase of the single gait cycle. * and ** show the images of minimum and maximum meniscus extrusion during walking (A). The representative waveform of meniscus extrusion during walking (B). The waveform was constructed by the sequenced value of meniscus extrusion in about 20 images and was shown time-normalized to the 101 data points of a single stance phase. The arrow shows ∆MME (1.6 mm)

In the dynamic condition, around 20 images were obtained for a trial, and the minimum and maximum MMEs from these images were determined. Additionally, the ΔMME was defined as the difference between the minimum and maximum MME in the images (Fig. 3A,B). Moreover, it made the waveform of meniscus extrusion during walking from the continual values of the MME and was time-normalized to the 101 data points of a single stance phase. The timing of the peak in waveform was detected in each participant (Fig. 3B).

**Fig. 4:**
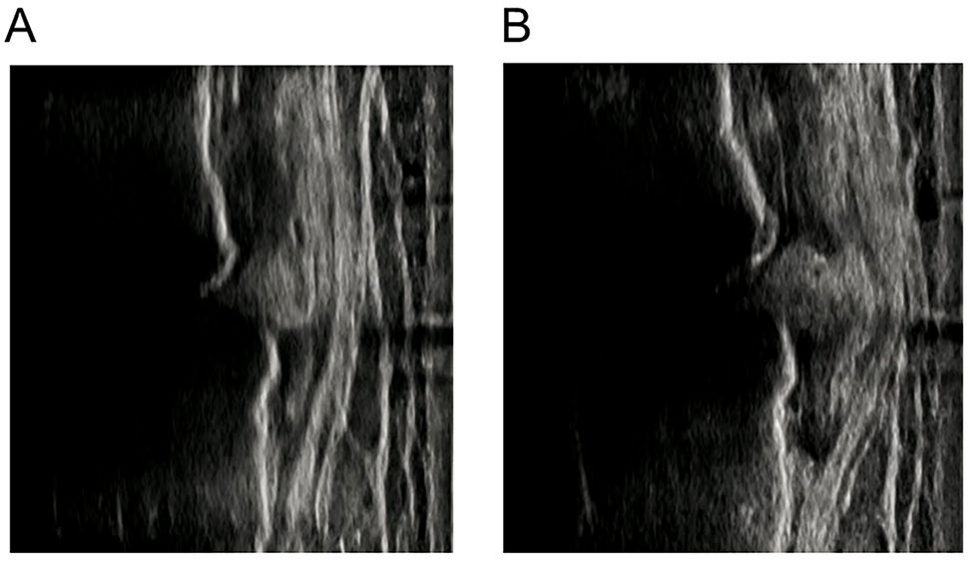
Representative images of the extruded menisci in different static positions. Supine (A) and unipedal standing (B) and it calculates the ∆MME (0.7 mm)

In contrast, in the static condition the MME was acquired from supine and unipedal standing positions (Fig. 4). The values of the MMEs in the supine and unipedal standing positions were adopted for comparison with the minimum and maximum MMEs in the dynamic condition. The ΔMME was defined as the difference in the MME between the supine and unipedal standing positions. The representative value was the average value from three measurements and was used for statistical analyses.

### Statistical analysis

For comparing the conditions, the mean values of the minimum and maximum MMEs were calculated by two-way factorial analysis of variance with repeated measures in each condition. Moreover, the ΔMME was compared using the Wilcoxon signed-rank test. MMPRT directly led to the destruction of the menisci hoop function and the presence of MMPRT was used to create subgroups. To compare the demographic data, knee pain, knee adduction moment, MME, and ΔMME for the MMPRT subgroups, the Mann–Whitney U Test was performed. Pearson’s or Spearman’s correlation methods were used to determine the correlation with knee pain in each condition or subgroup, respectively. Statistical analyses were performed using SPSS (v23, IBM, USA), and the critical value for significance was set at *P* < 0.05.

Moreover, the power analysis was performed by G*power, and showed a power of 0.88 to detect significant correlations between knee pain and ΔMME with the current sample size.

## Results

### Participant demographic data and meniscus quality

The participants had mild and moderate knee OA with varus alignment. The demographic data are summarized in Table 1.

**Table 1:** Demographic data of the participants

	Knee OA	MMPRT	no-MMPRT	*P*
N / knees	32 / 32	8 / 8	24 / 24	
K/L (I, II, III)	4, 17, 11	2, 4, 2	2, 13, 9	
Sex (M: F)	13: 19	2: 6	11: 13	
Age (years)	60.5 ± 9.9	62.1 ± 6.8	60.0 ± 10.6	0.53
BMI (kg/m²)	24.4 ± 3.2	25.2 ± 2.7	24.2 ± 3.3	0.4
FTA (°)	179.8 ± 3.4	178.7 ± 3.4	180.1 ± 3.3	0.33

K/L, Kellgren-Lawrence grade; BMI, Body mass index; FTA, femorotibial angle; MMPRT, medial meniscus posterior root tear. Values represent means ± standard deviations. The *P*-value shows the difference between the groups with and without MMPRT.

An abnormal medial meniscus was confirmed in 30 (94%) knees of patients with OA through MRI. The type of meniscus tear and the complexity were analyzed and 15 (47%) had complex tears in this study. In contrast, MMPRT was present in eight (25%). In terms of location, the abnormal sections were approximately equal except for the middle section, which was less frequent (Table 2).

**Table 2:** Type and location of meniscal tears from MRI

**Type of tear**	**n = 32**
Normal intensity	2 (6)
Longitudinal tear	2 (6)
Horizontal tear	5(16)
Complex tear	15 (47)
MMPRT	8 (25)
**Location of tear**	**n = 30**
Middle	3 (10)
Middle to posterior	9 (30)
Posterior	10 (33)
Posterior root	8 (27)

Values represent n (%). MMPRT, medial meniscus posterior root tear

### Comparison of MME and ΔMME for static and dynamic conditions

**Fig. 5:**
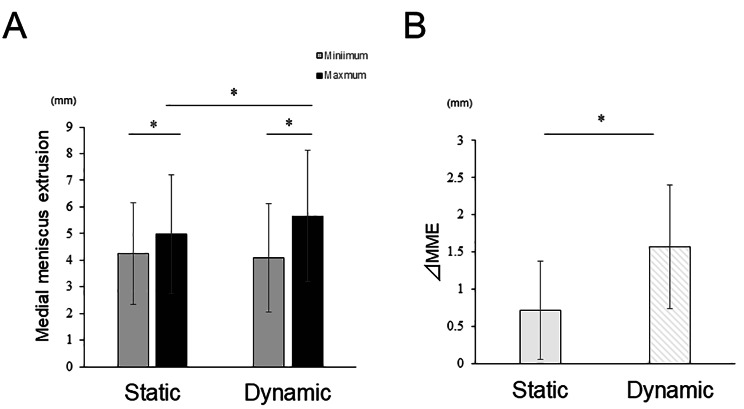
Comparison of the MME and ΔMME in each condition. MME (A) and ΔMME (B). MME, medial meniscus extrusion; ΔMME, the difference in MME between maximum and minimum within the condition. The MMEs in the supine and unipedal standing positions were adopted for comparison with the minimum and maximum. Values represent means ± standard deviations. * shows the significant differences between groups or conditions (*P* < 0.05)

There was no significant difference in the minimum MME for both conditions (static: 4.2 ± 1.9 mm, dynamic: 4.1 ± 2.0 mm). However, in the dynamic condition, the maximum MME was significantly higher than that in the static condition (static: 4.9 ± 2.2 mm, dynamic: 5.6 ± 2.4 mm; *P* < 0.05) **(**Fig. 5A**)**. Additionally, the ΔMME in the dynamic condition was significantly higher than that in static condition (static:0.7 ± 0.6 mm, dynamic:1.5 ± 0.8 mm; *P* < 0.01) **(**Fig. 5B**)**.

### Walking parameters and dynamic MME

The walking speed and cadence were 0.88 ± 0.13 m/s and 108.1 ± 10.6 step/min.

The MME during walking gradually increased in the stance phase, and the peak-timing of MME (maximum MME) was 70.8 ± 18.8%, which showed mid to late in the stance phase of the gait cycle.

### Correlations between knee pain, biomechanical data, and ΔMME in each condition

The mean value of pain on the VAS was 37.9 ± 22.7 mm. The first and second moment peaks of knee adduction were 0.5 ± 0.2 and 0.4 ± 0.2 Nm/kg and peak timings were 30.4 ± 5.8 and 69.9 ± 6.2% in the stance phase of the gait cycle.

In the static condition, no significant correlations between ΔMME and VAS and knee adduction moments were observed (Table 3). In contrast, in the dynamic condition, the ΔMME had significant positive correlations with VAS (r = 0.55; *P* < 0.01) and the second knee adduction moment peak (r = 0.44; *P* < 0.05) (Table 3). However, there was no correlation between VAS and the knee adduction moment peak.

**Table 3:** Correlations between ΔMME in each condition

	Static	Dynamic
VAS	0.22	0.55*
First knee adduction moment	0.07	0.21
Second knee adduction moment	0.29	0.44*

ΔMME, is the difference in medial meniscus extrusion between the maximum and minimum within the condition. VAS, visual analog scale. Values represent a correlation coefficient with ΔMME and * is the significant correlation (*P* < 0.05)

### Comparison between the ΔMME under the dynamic condition for the MMPRT subgroup

There were no significant differences in the demographic data, VAS, or the minimum and maximum MMEs for the subgroups (Tables 1 and 4). However, the ΔMME was significantly lower than that in the no-MMPRT subgroup (MMPRT:1.1 ± 0.4 mm, no-MMPRT:1.7 ± 0.9 mm; *P* < 0.05) (Table 4).

**Table 4:** Comparison between VAS, MME, and ΔMME during walking in the MMPRT subgroups

	MMPRT	no-MMPRT	*P*	
VAS (mm)	35.0 ± 23.8	37.1 ± 22.7	0.83	
Minimum MME (mm)	3.6 ± 1.1	4.2 ± 2.1	0.31	
Maximum MME (mm)	4.7 ± 0.9	5.9 ± 2.6	0.085	
ΔMME (mm)	1.1 ± 0.4	1.7 ± 0.9	0.035	

MMPRT, medial meniscus posterior root tear; VAS, visual analog scale; MME, medial meniscus extrusion; ΔMME, the difference in medial meniscus extrusion between maximum and minimum during walking. The values represent the means ± standard deviations. The *P*-value shows the difference between the with and without MMPRT groups

The VAS had significant positive correlations with ΔMME in both subgroups (MMPRT: r = 0.78, *P* < 0.05, no-MMPRT: r = 0.54, *P* < 0.01).

## Discussion

Our study demonstrated that the behavior of the MME in the dynamic evaluation was higher than that in static evaluation, and that the behavior of the MME was correlated with knee pain in patients with mild to moderately severe knee OA.

Our data revealed that the ΔMME in dynamic evaluation correlated with the pain VAS score, but that in static evaluation did not. Additionally, the dynamic evaluation detected sensitive knee pain according to menisci disfunction and supported our hypothesis. Knee pain often presents in dynamic situations, and depends on the mechanical stress in the knee. A previous study compared mechanical stress in the knee between static and dynamic conditions. They concluded that the mechanical stress during walking was 2–3 times higher than that experienced during static standing [13]. Additionally, the behavior of the MME also depends on the amount of mechanical stress in the medial compartment of the knee [6, 18–21]. In this study, the maximum MME evaluated during walking was higher than that in unipedal standing, and the peak-timing of MME was similar to that in the adduction moment of the second knee. This correlated significantly with the ΔMME. The results of these previous studies, combined with our results, explains the plausible correlation between knee pain and the behavior of the MME according to mechanical stress on the medial compartment in the dynamic condition.

For patients with MMPRT, there was no difference in the value of the MME itself; the ΔMME indicated that the behavior of the MME was lower than that in no-MMPRT. The MMPRT is known to directly lead to the destruction of the menisci hoop function and is one of the factors for worsening of the MME [22–24]. However, its value of MME directly depends on the severity of the knee OA [6, 25]. Our study included several participants in different K/L stages, and it might explain the masking effect of the type of meniscus tear on the MME itself. However, the behavior of the MME reflects the destruction of the hoop function and its extreme reaction often presents in patients with knee OA [26–28]. Karpinski et al. reported on menisci behavior in MMPRT. Their results showed that patients with MMPRT presented with poor meniscus behavior. This is indicative of an absent hoop reaction due to destruction of the meniscus structure [29]. The behavior of the MME in knee OA is different with and without MMPRT. In this study, MMPRT was strongly correlated with knee pain compared to its absence. Therefore, in cases with absent menisci hoop function, the behavior of the MME may demand extreme work for a meniscus and is a feature of symptomatic knee OA.

Interestingly, our data showed that knee pain was correlated with ΔMME, but not in the second moment peak which had similar peak-timing and correlation with ΔMME. Typically, the ΔMME reflects the instability of the meniscus when loading stress [30]. Moreover, greater ΔMME is a feature of early progression of knee OA and the intensity of knee pain according to the bone marrow lesion, in which the behavior of the MME is a critical indicator of the contact pressure in the tibiofemoral joint in the knee [6, 8, 27, 28]. In contrast, the knee adduction moment is known to be the mechanical stress on the medial compartment of the knee [31, 32]. The knee adduction moment is not a direct indicator of contact pressure in the medial compartment if the participant has a pathological meniscus. Often no relationship is seen between changes in the knee adduction moment and knee pain after interventions [33–35]. In this study, our data also showed that the knee adduction moment had no correlation with knee pain, and that there was only a small correlation with ΔMME. Therefore, these results, and those of previous studies, show that the knee adduction moment itself might not directly explain knee pain that depends on contact pressure.

However, sensitive detection of true mechanical stress is important for knee OA. In a previous study, mechanical stress was measured during walking, on the other hand the behavior of the menisci were measured static condition [6]. Therefore, intraarticular tissue reaction upon mechanical stress is difficult to be evaluated and the mechanism of knee pain remains not elucidated. The dynamic ultrasound technique can demonstrate time synchronized ultrasound with three-dimensional motion analysis and provide real-time information of the intraarticular tissue response to mechanical stress. Interestingly, tissue response upon mechanical stress were different in each patient with knee OA. Of note, it was demonstrated that the type of meniscal tear affect behavior of meniscus. Therefore, evaluation of meniscal behavior during walking with dynamic ultrasound technique may help understand the mechanism of knee pain and patients status. In addition, it may also useful to evaluate the efficacy of interventions, such as lateral wedge insoles [12], high tibia osteotomy [36], and root repair [37], which are based on evidence that they reduce knee pain by controlling MME or knee adduction moments.

The present study had several limitations. First, we cannot indicate that the behavior of the meniscus causes painful knees because of the cross-sectional nature of the study. Future studies need to confirm the correlation between knee pain and the behavior of the meniscus in a longitudinal design. Second, we did not have a control group with patients with asymptomatic knee OA to compare the MME and behavior of the meniscus. Third, we did not have a sufficient number of patients in the subgroups. This may lead to insufficient analysis as it did not show the cut off value and the effect of the type of MMPRT on the behavior of the meniscus [38]. A further longitudinal study is needed with a control group and inclusion of a large sample of patients with MMPRT.

## Conclusion

The conventional static evaluation could underestimate the response to loading stress onto the meniscus. The dynamic approach may be a valid evaluation tool for understanding the mechanism of knee pain and sensitively detecting the features of symptomatic knee OA.

## Data Availability

The datasets generated during, and/or analyzed during, the current study are available from the corresponding author on reasonable request.
